# Predictors of death or lung transplant after a diagnosis of idiopathic pulmonary fibrosis: insights from the IPF-PRO Registry

**DOI:** 10.1186/s12931-019-1043-9

**Published:** 2019-05-30

**Authors:** Laurie Snyder, Megan L. Neely, Anne S. Hellkamp, Emily O’Brien, Joao de Andrade, Craig S. Conoscenti, Thomas Leonard, Shaun Bender, Mridu Gulati, Daniel A. Culver, Robert J. Kaner, Scott Palmer, Hyun Joo Kim, on behalf of the IPF-PRO™ Registry investigators, Wael Asi, Albert Baker, Scott Beegle, John A. Belperio, Rany Condos, Francis Cordova, Daniel A. Culver, Joao A. M. de Andrade, Daniel Dilling, Kevin Flaherty, Marilyn Glassberg, Mridu Gulati, Kalpalatha Guntupalli, Nishant Gupta, Amy Hajari Case, David Hotchkin, Tristan Huie, Robert Kaner, Hyun Kim, Maryl Kreider, Lisa Lancaster, Joseph Lasky, David Lederer, Doug Lee, Timothy Liesching, Randolph Lipchik, Jason Lobo, Yolanda Mageto, Prema Menon, Lake Morrison, Andrew Namen, Justin Oldham, Rishi Raj, Murali Ramaswamy, Tonya Russell, Paul Sachs, Zeenat Safdar, Barry Sigal, Leann Silhan, Mary Strek, Sally Suliman, Jeremy Tabak, Rajat Walia, Timothy P. Whelan, Julie Fleming, Wendy Morris

**Affiliations:** 1 Duke Clinical Research Institute, Durham, NC USA; 2 Duke University Medical Center, Durham, NC USA; 3 University of Alabama at Birmingham, Birmingham, AL USA; 4 Boehringer Ingelheim Pharmaceuticals, Inc., Ridgefield, CT USA; 5 Yale School of Medicine, New Haven, CT USA; 6 Cleveland Clinic, Cleveland, OH USA; 7 Weill Cornell Medicine, New York, NY USA; 8 University of Minnesota, Minneapolis, MN USA

## Abstract

**Background:**

Idiopathic pulmonary fibrosis (IPF) is a progressive disease with a variable clinical course and high mortality. We used data from a large national US registry of patients with IPF to investigate relationships between patient characteristics, including markers of disease severity, and mortality.

**Methods:**

The analysis cohort comprised patients enrolled in the IPF-PRO Registry from its inception on 5 June 2014 to 26 October 2017. The primary criterion for inclusion in this registry is that patients must be diagnosed or confirmed with IPF at the enrolling centre within 6 months. Associations between patient characteristics and markers of disease severity at enrolment and mortality outcomes were investigated using univariable, multivariable and adjustment models.

**Results:**

Among 662 patients enrolled, 111 patients died or had a lung transplant over a follow-up period of 30 months. The probability of being free of both events at month 30 was 50.6% (95% CI: 40.0, 60.2). When patient characteristics and markers of disease severity were jointly examined in a multivariable analysis, oxygen use at rest (hazard ratio [HR] 2.44 [95% CI: 1.45, 4.10]), lower forced vital capacity (FVC) % predicted (HR 1.28 [95% CI: 1.10, 1.49] per 10% decrease) and diffusion capacity for carbon monoxide (DLco) % predicted (HR 1.25 [95% CI: 1.04, 1.51] per 10% decrease) were significantly associated with increased risk of death or lung transplant. The risk of death or lung transplant increased with increasing age in patients ≥62 years old (HR 1.18 [95% CI: 0.99, 1.40] per 5-year increase), and decreased with increasing age in patients <62 years old (HR 0.60 [95% CI: 0.39, 0.92] per 5-year increase).

**Conclusions:**

In an observational US registry of patients with IPF, oxygen use at rest, lower FVC % predicted, and lower DLco % predicted were associated with risk of death or lung transplant. An audio podcast of the lead author discussing these data can be downloaded from: http://www.usscicomms.com/respiratory/snyder/IPF-PROsurvival1/.

**Trial registration:**

ClinicalTrials.gov number: NCT01915511.

**Electronic supplementary material:**

The online version of this article (10.1186/s12931-019-1043-9) contains supplementary material, which is available to authorized users.

## Introduction

Idiopathic pulmonary fibrosis (IPF) is a progressive fibrosing interstitial lung disease (ILD) characterised by decline in lung function and high mortality [1]. IPF mainly affects older male adults, typically presenting in the sixth or seventh decade in individuals with a history of smoking [2]. Based on data collected in the US prior to the availability of antifibrotic therapy, median survival following diagnosis in patients with IPF was 3–5 years [2–4]. Similar mortality was observed in patients with IPF in a pan-European registry (eurIPFreg) who were not receiving antifibrotic therapy [5].

IPF has a variable clinical course, but a number of patient and clinical characteristics have been shown to be predictors of mortality in single-centre reports, clinical trial data and registry studies. These include older age; male sex; lower body mass index (BMI); definite usual interstitial pneumonia (UIP) pattern on high-resolution computed tomography (HRCT); low, or decline in, forced vital capacity (FVC), diffusing capacity of the lungs for carbon monoxide (DLco), or exercise capacity (6-min walk distance, 6MWD); use of supplemental oxygen; and a history of respiratory-related hospitalisation [4, 6–14]. Importantly, acute deteriorations in respiratory function, known as acute exacerbations, have a very poor prognosis [12, 15]. In-hospital mortality following an acute exacerbation is estimated to be over 50% [15].

The Idiopathic Pulmonary Fibrosis Prospective Outcomes (IPF-PRO) Registry (NCT01915511) is an ongoing observational US registry of patients diagnosed or confirmed with IPF at the enrolling centre within 6 months [16]. Unlike clinical trials, patients with any severity of disease are eligible to enter the IPF-PRO Registry. As such, the registry provides an opportunity to better understand factors associated with disease progression in a diverse, well-characterised cohort of patients with IPF. We conducted an in-depth analysis of patient characteristics and markers of disease severity at enrolment that were associated with death or lung transplant in patients with IPF.

## Methods

### Study cohort

Patients enrolled in the IPF-PRO Registry from its inception on 5 June 2014 to 26 October 2017 comprised the analysis cohort. The design of this registry has been described [16]. Participants are required to be diagnosed or confirmed with IPF at the enrolling centre within 6 months according to the 2011 ATS/ERS/JRS/ALAT guidelines [1]. Patients with malignancy (other than skin cancer) within the past 5 years, or who are listed for lung transplantation or participating in a randomised clinical trial, are not eligible to enrol in the registry; however, patients can join clinical trials or be listed for lung transplantation after enrolment.

### Outcome

The primary outcome was a composite of death or lung transplant. Lung transplant serves as a marker of disease progression that would otherwise have been expected to result in death. Secondary outcomes were death, a composite of respiratory-related death or lung transplant, and respiratory-related death. Deaths were recorded in case report forms. In addition, telephone interviews every 6 months confirmed patients’ vital status; if the patient had died, the date of death was obtained and entered into the registry database. Whether a death was respiratory-related was determined by the principal investigator at the site based on review of the medical records surrounding the death.

### Statistical analysis

Cumulative event counts and event-free rates over 30 months (a cut-off selected based on available follow-up data) were estimated for each outcome using the Kaplan–Meier method. A Cox proportional hazards regression model for time-to-first-event was used in association analyses. The Cox model was stratified by antifibrotic drug use (nintedanib or pirfenidone) at enrolment, which allowed the baseline hazard function to vary across strata and assumed that the effect of the other covariates in the model was the same across strata. In this way, the stratified Cox model accounted for treatment use without directly reporting its effect estimate. Associations between patient characteristics at enrolment and each outcome were examined using univariable and multivariable models. Based on previous studies, clinical experience, and data completeness (variables with missing data from ≥25% of patients were not considered), the following covariates were evaluated as patient characteristics: age, sex, BMI, private insurance, smoking status, oxygen use with activity, oxygen use at rest, 2011 ATS/ERS/JRS/ALAT diagnostic criteria for IPF (definite, probable, possible) [1], history of coronary artery disease or congestive heart failure, history of pulmonary hypertension, clinically significant emphysema on HRCT scan (based on the opinion of the investigator), prior hospitalisation, distance to the enrolling centre, and time from symptom onset to confirmed diagnosis of IPF at the enrolling centre. Data on these covariates were abstracted from patients’ medical records. Univariable models included only one covariate. The multivariable model included all covariates. In addition, an adjustment model was created that included patient characteristics selected after performing backwards selection on the multivariable model using an alpha-to-stay criterion of 0.05; selection was performed to develop a parsimonious list of patient characteristics to use as adjustment covariates for association analyses between markers of disease severity and each outcome. Associations between each marker of disease severity and each outcome were examined using a univariable model and a model adjusted for patient characteristics selected in the adjustment model. The following covariates were evaluated as markers of disease severity: FVC % predicted, DLco % predicted, the number of prior respiratory-related hospitalisations, composite physiologic index (CPI) [17] and GAP stage [18].

The joint association between patient characteristics and markers of disease severity and death or lung transplant was then examined. This was assessed by including patient characteristics that were selected in the adjustment model and markers of disease severity that were significant predictors of death or lung transplant after adjustment for patient characteristics simultaneously in a multivariable model.

Categorical and continuous patient characteristics and markers of disease severity were assessed for appropriate distribution and linearity. For categorical variables, the distribution of events across levels was examined. If there were <5 events in some levels, levels of the categorical variable were collapsed before being included in the final association model. For continuous variables, the linearity assumption was assessed by performing a lack-of-fit test comparing a linear fit with a non-linear fit based on a restricted cubic spline with 3 knots. Only age showed a non-linear relationship; this was transformed to account for the non-linearity using a 2-part linear spline with the knot at 62 years, a point chosen based on the unadjusted model chi square. For all variables, the proportional hazards assumption was assessed by testing for a significant interaction between log-transformed time-to-event and the variable. Missing data were handled using multiple imputation (see Additional file 1).

## Results

### Study cohort

A total of 662 patients were included in this analysis. At enrolment, median age was 70 years, 74.9% of patients were male, 68.4% were current or former smokers and 19.6% were using supplemental oxygen at rest (Table 1). Median FVC was 69.6% predicted and median DLco was 41.7% predicted.

**Table 1:** Characteristics of patients at enrolment into the IPF-PRO Registry (*n* = 662)

Age, years	70 (65, 75)
Male	496 (74.9)
White	623 (94.1)
Body mass index, kg/m^2^	29.0 (26.0, 32.4)
Private insurance	420 (66.4)
Current or former smoker	446 (68.4)
Oxygen use with activity	217 (34.1)
Oxygen use at rest	125 (19.6)
Serologic testing reported at enrolment At least one abnormal serologic test reported	184 (27.8) 161
Receiving immunosuppressive or cytotoxic medications	5 (0.8)
Receiving nintedanib or pirfenidone	352 (54.0)
Surgical lung biopsy performed prior to enrolment	176 (30)
Bronchoscopy performed within 12 months of enrolment	60 (10.3)
Prior diagnosis of IPF (confirmed at the enrolling centre)	301 (45)
Diagnostic criteria^a^	
Definite IPF	437 (68.8)
Probable IPF	141 (22.2)
Possible IPF	57 (9.0)
History of coronary artery disease or congestive heart failure	203 (31.2)
History of pulmonary hypertension	51 (7.9)
Emphysema^b^	71 (11.2)
Prior hospitalisation (any)	171 (29.3)
Respiratory related	106 (18.2)
Non-respiratory related	86 (14.8)
Symptom onset to confirmed diagnosis of IPF at enrolling center, months	14 (7, 29)
Distance to enrolling centre, miles	40 (16, 109)
FVC, % predicted	69.6 (60.1, 79.9)
DLco, % predicted	41.7 (32.2, 50.1)
Prior respiratory hospitalisation	106 (18.2)
1	84 (12.7)
2	18 (2.7)
3	3 (0.5)
4	1 (0.2)
Composite physiologic index	53.2 (45.7, 60.0)
GAP stage	
I	146 (26.4)
II	306 (55.2)
III	102 (18.4)

Data are median (25th, 75th percentile) or n (%). Not all patients provided data on all variables. ^a^According to 2011 ATS/ERS/JRS/ALAT diagnostic guidelines [1]. ^b^Clinically significant emphysema on HRCT scan (based on the opinion of the investigator)

### Events of death or lung transplant

**Fig. 1:**
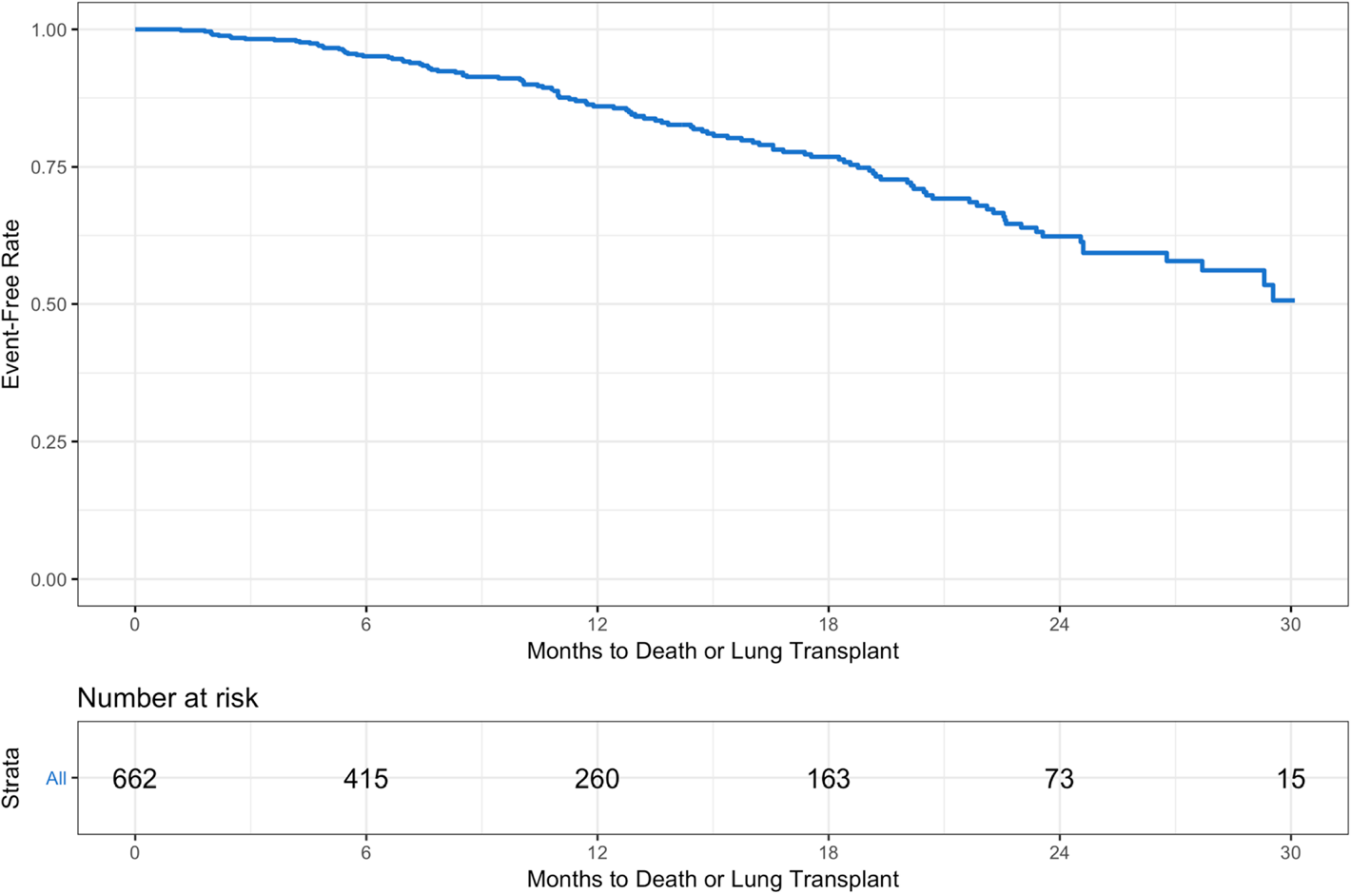
Kaplan-Meier estimate of time from enrolment in the IPF-PRO Registry to death or lung transplant

A total of 92 deaths and 20 lung transplants were observed, with 91 deaths and 20 lung transplants by month 30 used in the analysis. The event-free probability at month 30 was 50.6% (95% CI: 40.0, 60.2) (Fig. 1; Table 2). Cumulative event counts and event-free rates for death, respiratory-related death or lung transplant, and respiratory-related death are shown in Additional file 2: Table S1 and Additional file 3: Figures S1–S3.

**Table 2:** Events of death or lung transplant

	Cumulative event count	Number of patients at risk	Event-free probability, % (95% CI)
Month 6	24	415	95.1 (92.8, 96.7)
Month 12	57	260	86.0 (82.1, 89.0)
Month 18	80	163	76.8 (71.7, 81.1)
Month 24	104	73	62.3 (55.4, 68.5)
Month 30	111	15	50.6 (40.0, 60.2)

### Associations between patient characteristics and outcomes

**Fig. 2:**
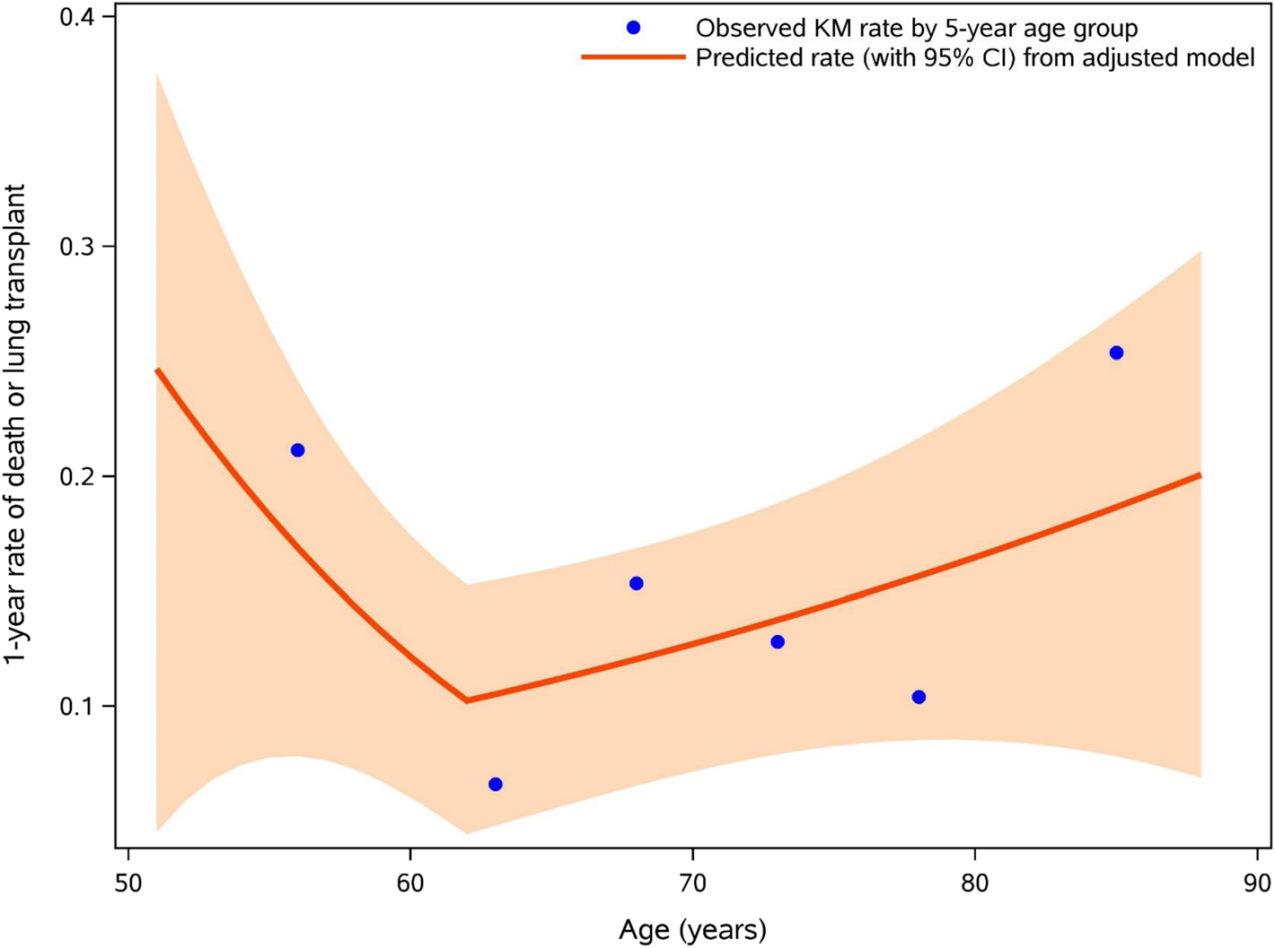
Spline transformation for patient age at enrolment. Orange line and band show predicted 1-year event rate with 95% CI from the model including age, oxygen use at rest, oxygen use with activity, FVC % predicted and DLco % predicted. Observed (Kaplan-Meier) rates are from age groups defined in 5-year intervals. Group age ranges and patient numbers are: ≤60 years (*n* = 76), 61–65 years (*n* = 99), 66–70 years (*n* = 175), 71–75 years (*n* = 165), 75–80 years (*n* = 103) and >80 years (*n* = 44)

As a J-shaped relationship was observed between age and death or lung transplant, this was modelled using a 2-part linear spline with a single knot at 62 years (Fig. 2). There was no evidence of non-linearity for the other continuous patient characteristics assessed (data not shown).

**Fig. 3:**
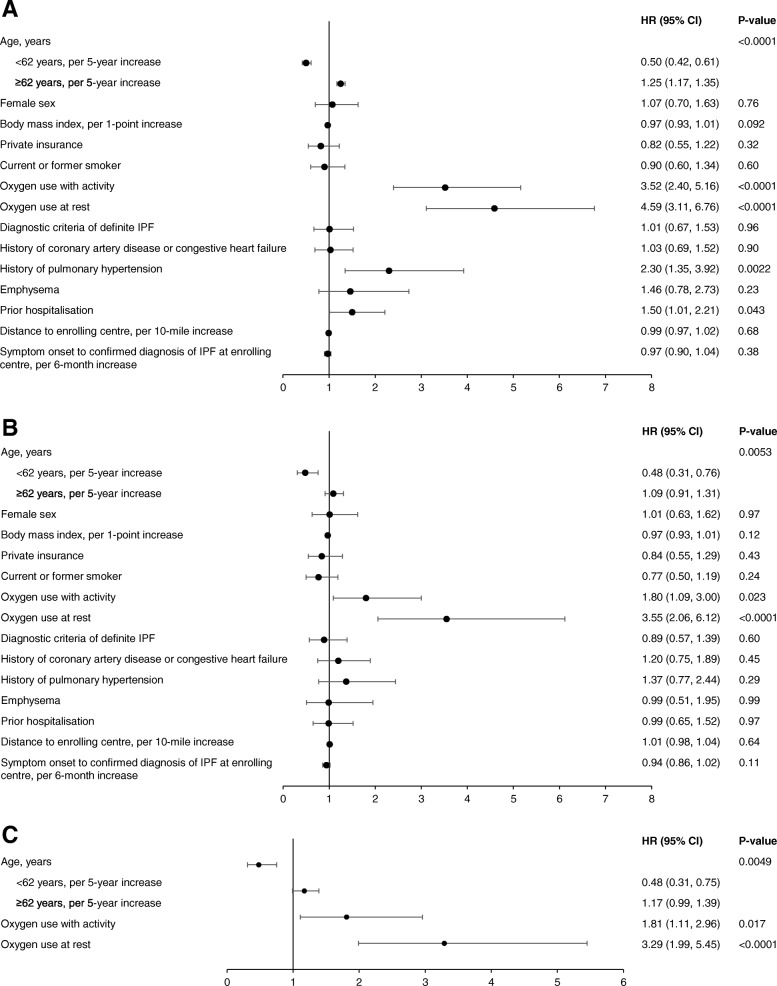
Associations between patient characteristics and death or lung transplant in (**a**) univariable models, **b** a multivariable model, and (**c**) an adjustment model

In the univariable analyses, oxygen use with activity (HR 3.52 [95% CI: 2.40, 5.16]), oxygen use at rest (HR 4.59 [95% CI: 3.11, 6.76]), history of pulmonary hypertension (HR 2.30 [95% CI: 1.35, 3.92]) and prior hospitalisation (HR 1.50 [95% CI: 1.01, 2.21]) were significantly associated with an increased risk of death or lung transplant (Fig. 3a). The risk of death or lung transplant increased for every 5-year increase in age in patients ≥62 years old (HR 1.25 [95% CI: 1.17, 1.35]), and increased per 5-year decrease in age in patients <62 years old (HR 0.50 [95% CI: 0.42, 0.61]).

In the multivariable analysis, oxygen use with activity (HR 1.80 [95% CI: 1.09, 3.00]), and oxygen use at rest (HR 3.55 [95% CI: 2.06, 6.12]) were significantly associated with an increased risk of death or lung transplant (Fig. 3b). The risk of death or lung transplant increased numerically with increasing age in patients ≥62 years old (HR 1.09 [95% CI: 0.91, 1.31] per 5-year increase), and decreased significantly with increasing age in patients <62 years old (HR 0.48 [95% CI: 0.31, 0.76] per 5-year increase). When backwards selection was performed using the covariates from the multivariable model, the same three variables were significantly associated with this outcome (Fig. 3c).

Associations between patient characteristics and death, respiratory-related death or lung transplant, and respiratory death were generally consistent with the data on death or lung transplant (Additional file 2: Tables S2–S4).

### Associations between markers of disease severity and outcomes

**Fig. 4:**
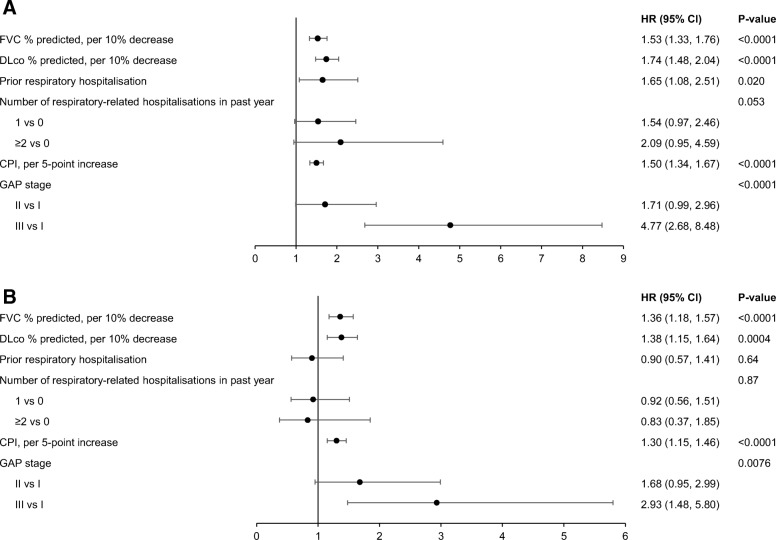
Associations between markers of disease severity and death or lung transplant in (**a**) univariable models and (**b**) a model adjusted for patient characteristics

There was no evidence of non-proportional hazards or non-linearity for any of the markers of disease severity assessed (data not shown). In the univariable analyses, prior respiratory-related hospitalisations (HR 1.65 [95% CI: 1.08, 2.51]), worse FVC % predicted (HR 1.53 [95% CI: 1.33, 1.76] per 10% decrease), worse DLco % predicted (HR 1.74 [95% CI: 1.48, 2.04] per 10% decrease), worse disease severity according to CPI (HR 1.50 [95% CI: 1.34, 1.67] per 5-point increase) and GAP stage (HR 1.71 [95% CI: 0.99, 2.96] for II vs I; HR 4.77 [95% CI: 2.68, 8.48] for III vs I) were significantly associated with an increased risk of death or lung transplant (Fig. 4a). In the analysis adjusted for patient characteristics in the adjustment model (i.e., age, oxygen use with activity, oxygen use at rest), worse FVC % predicted (HR 1.36 [95% CI: 1.18, 1.57] per 10% decrease), worse DLco % predicted (HR 1.38 [95% CI: 1.15, 1.64] per 10% decrease), and worse disease severity according to CPI (HR 1.30 [95% CI: 1.15, 1.46] per 5-point increase) and GAP stage (HR 1.68 [95% CI: 0.95, 2.99] for II vs I; HR 2.93 [95% CI: 1.48, 5.80] for III vs I) were significantly associated with an increased risk of death or lung transplant (Fig. 4b). Associations between markers of disease severity and death, respiratory-related death or lung transplant, and respiratory death were generally consistent with the data on death or lung transplant (Additional file 2: Tables S5–S7). Comparison of markers of disease severity between those with a new diagnosis of IPF and those referred with a diagnosis of IPF is presented in Additional file 2: Table S8.

### Joint associations between patient characteristics and markers of disease severity and outcome

**Fig. 5:**
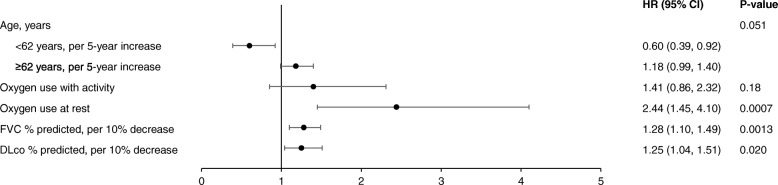
Joint association between patient characteristics and disease severity markers, and death or lung transplant

When patient characteristics in the adjustment model and markers of disease severity that were significant predictors of death or lung transplant after adjustment (except for CPI and GAP stage, as these composites include FVC and DLco) were included simultaneously in a multivariable model, oxygen use at rest (HR 2.44 [95% CI: 1.45, 4.10]), worse FVC % predicted (HR 1.28 [95% CI: 1.10, 1.49] per 10% decrease) and lower DLco % predicted (HR 1.25 [95% CI 1.04, 1.51] per 10% decrease) were significantly associated with increased risk of death or lung transplant (Fig. 5). The risk of death or lung transplant increased numerically with increasing age in patients ≥62 years old (HR 1.18 [95% CI: 0.99, 1.40] per 5-year increase), and decreased with increasing age in patients <62 years old (HR 0.60 [95% CI: 0.39, 0.92] per 5-year increase). Oxygen use at rest was the strongest predictor of death or lung transplant (largest test-statistic value in the regression modelling). Joint associations between patient characteristics and disease severity markers, and death, respiratory-related death or lung transplant, and respiratory death are summarised in Additional file 2: Table S9.

## Discussion

As IPF is a progressive disease associated with high mortality, there is considerable interest in defining patient characteristics or markers of disease severity that associate with an increased risk of mortality or lung transplant. In a national US registry of patients diagnosed or confirmed with IPF at the enrolling centre within 6 months, we found that the probability of death or lung transplant over a follow-up period of 30 months, based on Kapan-Meier estimates, was approximately 50%. The variable that was most strongly associated with the risk of death or lung transplant was the use of supplemental oxygen at rest, reported in 20% of patients at enrolment. Interestingly, of the patients on oxygen at rest, only 41% had a diagnosis of IPF prior to being referred to the enrolling centre. Based on the limited arterial blood gas measurements (13% of this cohort) reported as part of clinical care in the registry, we cannot determine if there were more patients who may have qualified for oxygen at rest but were not reported as being on oxygen at rest. In a comparison of those reported to be or not to be on oxygen at rest, the patients on oxygen at rest had a higher CPI and more advanced GAP stage (Additional file 2: Table S10). Regardless, oxygen use at rest was an important predictor of death or lung transplant across univariable analyses, multivariable analyses, and analyses adjusted for other patient characteristics or markers of disease severity. While this finding is consistent with shorter studies [8, 9, 19], confirmation in a large and diverse cohort of patients in the IPF-PRO Registry highlights the significance of this clinical characteristic as a predictor of mortality in patients with IPF in the real world. In addition, use of supplemental oxygen during activity was a predictor of death or lung transplant in the univariable, multivariable and adjusted models. This is consistent with previous observations that oxygen desaturation during exercise is a predictor of mortality in patients with IPF [11, 20]. However, oxygen use during activity was not as strong a predictor of death or lung transplant as oxygen use at rest.

Decline in FVC is reflective of disease progression in patients with IPF and a predictor of mortality [1]. Lower FVC % predicted at baseline has been associated with an increased risk of mortality in patients with IPF both in clinical trials [12] and in registry studies [11, 14, 21, 22]. In our analysis, the risk of death or lung transplant increased by 28% per 10% decrease in FVC % predicted at enrolment. Similarly, the risk of death or lung transplant increased by 25% per 10% decrease in DLco % predicted at enrolment. Importantly, these relationships persisted after adjusting for age and oxygen use.

We demonstrated a J-shaped relationship between age and death or lung transplant, with patients approximately 60 years of age being at the lowest risk, a gradual increase in risk with increasing age and a sharp increase in risk in younger patients. In our cohort, 91 patients (13.7%) were aged under 62 years and 18 patients (2.7%) were aged under 55 years. A greater proportion of patients aged under 55 years reported a family history of ILD than patients aged 62 years and older (35% versus 18%). We hypothesise that the youngest patients in the IPF-PRO Registry may represent a different disease cohort than the elderly patients, as familial pulmonary fibrosis has been associated with high mortality [23–25]. However, in a recent single-centre study of 129 patients with IPF, there was no significant difference in mortality over 3 years in patients aged under 50 years (*n* = 30) compared with older patients [26].

Previous analyses of data from clinical trials [12, 27] and retrospective studies [28–30] have linked respiratory-related hospitalisation with increased mortality in patients with IPF. In our analysis, prior respiratory-related hospitalisation was associated with an increased risk of death or lung transplant in univariable but not multivariable analyses. Previous studies have suggested that patients with idiopathic interstitial pneumonia and definite UIP have a worse prognosis than those with possible UIP [13, 31]. In our analyses, a site-confirmed diagnosis of definite IPF showed no association with the risk of death or lung transplant. This parallels data from the INPULSIS trials, which showed that patients with possible UIP and traction bronchiectasis on HRCT had the same rate of disease progression over 1 year as patients with honeycombing confirmed on HRCT or surgical lung biopsy [32].

Recently, Torrisi et al. reported a TORVAN model and index that incorporates comorbidities into a survival model. Specifically, worse survival was associated with pulmonary hypertension, lung cancer, valvular heart disease and atrial arrhythmias. Gastro-oesophageal reflux disease (GERD; by history and medication use) was protective in survival models. Given this recent report, we reviewed the data from the IPF-PRO Registry for the comorbidities of interest. Of note, pulmonary hypertension was found not to be associated with death or lung transplant in our cohort, after adjusting for other patient characteristics. There were no patients with lung cancer. We did not collect any information on valvular heart disease so cannot provide those data. Regarding atrial arrhythmias, there were 68 patients (10.5%) with a reported atrial fibrillation or atrial flutter history. For GERD, there were 459 patients (73%) with GERD (by history, proton pump inhibitor use, or H2 antagonist use) at time of enrolment. Given the small number of atrial arrhythmias, we do not have enough data to evaluate the predictive value of this variable. As the GERD variable was more prevalent, we included that in both the univariable and multivariable analyses for all four endpoints studied and it was not significant in any analysis. Further, it was not selected for the adjustment model for any endpoint. Thus, in our registry, we are not able to validate the TORVAN model, though it is likely that comorbidities may impact outcomes in some cohorts.

Our analyses of data from the IPF-PRO Registry have several strengths that distinguish them from other analyses of survival in patients with IPF. Firstly, the use of a large cohort of patients with IPF (*n* = 662) from many centres, recruited using broad inclusion criteria, emphasises the generalisability of our study in the real world. Secondly, the registry systematically collected data on a large number of covariates with high rates of follow-up, and was of longer duration than most clinical trials. There are also several limitations to note. Given the small number of lung transplants, we could not assess predictors of lung transplant as a stand-alone endpoint. Furthermore, we cannot confirm if older age in this cohort limited the number of lung transplants. We were limited in the analysis of associations between 6MWD at enrolment and death or lung transplant because many patients did not perform a 6-min walk test at enrolment. Due to the timing of the registry inception and the approval of nintedanib and pirfenidone in the US, we were not able to evaluate associations between the use of antifibrotic therapy at enrolment and death or lung transplant. Finally, our analyses were limited to patient characteristics at enrolment. In future, as the IPF-PRO Registry matures, it will provide an opportunity to assess novel measures of disease severity, such as quantitative lung fibrosis scores, and genetic, proteomic and metabolomic biomarkers of disease progression.

## Conclusions

Data from the IPF-PRO Registry demonstrated high mortality in patients with IPF, with oxygen use at rest being the strongest predictor of mortality over the follow-up period. Notably, the association between oxygen use at rest and death or lung transplant was independent of many factors previously associated with disease progression, such as FVC, DLco, GAP stage and hospitalisation. As such, careful consideration of oxygen requirements in patients with newly diagnosed IPF is a powerful prognostic tool to inform physician and patient decision-making regarding clinical care and potential treatment options.

## Additional files

Additional file 1: Handling of missing data. (DOCX 39 kb)

Additional file 2: Supplemental tables. (DOCX 63 kb)

Additional file 3: Supplemental figures. (DOCX 299 kb)

## Abbreviations

6MWD: 6-min walk distance
BMI: Body mass index
CPI: Composite physiologic index
DLco: Diffusing capacity of the lungs for carbon monoxide
FVC: Forced vital capacity
GAP: Gender, age, physiology
GERD: Gastro-oesophageal reflux disease
HR: Hazard ratio
HRCT: High-resolution computed tomography
ILD: Interstitial lung disease
IPF: Idiopathic pulmonary fibrosis
UIP: Usual interstitial pneumonia
