# Supplementary material for: Predictors of death or lung transplant after a diagnosis of idiopathic pulmonary fibrosis: insights from the IPF-PRO Registry
Source: Respir Res. 2019 May 30;20:105. doi: 10.1186/s12931-019-1043-9 (PMC6542049; doi:10.1186/s12931-019-1043-9)
Supplement: Supplementary file 1 — Handling of missing data. (DOCX 39 kb) [file 12931_2019_1043_MOESM1_ESM.docx]

**Handling of missing data**

Variables with missing data from ≥25% of patients were excluded from these analyses. Multiple imputation was implemented on the candidate predictor variables. Firstly, the missing data were filled in five times to generate five complete data sets as per the Full Conditional Specification method. Secondly, the five complete data sets were analysed using standard statistical analyses. Lastly, the results from the five complete datasets were combined to produce the final inferential results.
