# Supplementary material for: Predictors of death or lung transplant after a diagnosis of idiopathic pulmonary fibrosis: insights from the IPF-PRO Registry
Source: Respir Res. 2019 May 30;20:105. doi: 10.1186/s12931-019-1043-9 (PMC6542049; doi:10.1186/s12931-019-1043-9)
Supplement: Supplementary file 2 — Supplemental tables. (DOCX 63 kb) [file 12931_2019_1043_MOESM2_ESM.docx]

**Table S1.** Cumulative event counts and event-free probabilities for death, respiratory-related death or lung transplant, and respiratory-related death

|  | Cumulative event count | Number of patients at risk | Event-free probability,  % (95% CI) |
| --- | --- | --- | --- |
| **Death** |  |  |  |
| Month 6 | 20 | 460 | 96.3 (94.4, 97.6) |
| Month 12 | 45 | 310 | 90.4 (87.3, 92.8) |
| Month 18 | 65 | 195 | 83.8 (79.7, 87.2) |
| Month 24 | 85 | 93 | 73.8 (68.0, 78.7) |
| Month 30 | 91 | 22 | 65.4 (56.2, 73.1) |
| **Respiratory-related death or lung transplant** |  |  |  |
| Month 6 | 19 | 415 | 96.1 (94.0, 97.5) |
| Month 12 | 49 | 260 | 87.6 (83.9, 90.6) |
| Month 18 | 68 | 163 | 80.0 (75.1, 84.1) |
| Month 24 | 88 | 73 | 67.4 (60.6, 73.3) |
| Month 30 | 93 | 15 | 57.4 (46.1, 67.1) |
| **Respiratory-related death** |  |  |  |
| Month 6 | 15 | 460 | 97.2 (95.5, 98.3) |
| Month 12 | 37 | 310 | 91.9 (89.0, 94.1) |
| Month 18 | 53 | 195 | 86.6 (82.6, 89.7) |
| Month 24 | 69 | 93 | 78.4 (72.9, 82.9) |
| Month 30 | 73 | 22 | 71.4 (61.9, 78.9) |

**Table S2.** Associations between patient characteristics and death in univariable models, multivariable model and adjustment model

|  | Univariable models | | Multivariable model | | Adjustment model | |
| --- | --- | --- | --- | --- | --- | --- |
|  | HR (95% CI) | p-value | HR (95% CI) | p-value | HR (95% CI) | p-value |
| Age, years |  | <0.0001 |  | 0.037 |  | 0.0043 |
| <62 years, per 5-year increase | 0.46 (0.37, 0.56) |  | 0.53 (0.32, 0.88) |  | 0.51 (0.32, 0.83) |  |
| ≥62 years, per 5-year increase | 1.39 (1.29, 1.50) |  | 1.20 (0.99, 1.45) |  | 1.30 (1.10, 1.55) |  |
| Female sex | 1.00 (0.62, 1.60) | 0.99 | 0.99 (0.60, 1.65) | 0.98 | --- |  |
| Body mass index, per 1-point increase | 0.96 (0.92, 1.01) | 0.091 | 0.97 (0.93, 1.02) | 0.23 | --- |  |
| Private insurance | 0.73 (0.47, 1.12) | 0.15 | 0.73 (0.45, 1.18) | 0.19 | --- |  |
| Current or former smoker | 0.92 (0.59, 1.42) | 0.71 | 0.75 (0.47, 1.21) | 0.24 | --- |  |
| Oxygen use with activity | 3.52 (2.31, 5.39) | <0.0001 | 1.82 (1.02, 3.27) | 0.044 | 1.81 (1.03, 3.18) | 0.038 |
| Oxygen use at rest | 4.63 (3.01, 7.10) | <0.0001 | 3.05 (1.67, 5.55) | 0.0003 | 2.93 (1.67, 5.14) | 0.0002 |
| Diagnostic criteria of definite IPF | 0.96 (0.61, 1.52) | 0.87 | 0.88 (0.54, 1.44) | 0.61 | --- |  |
| History of coronary artery disease or congestive heart failure | 1.17 (0.76, 1.80) | 0.47 | 1.23 (0.75, 2.00) | 0.41 | --- |  |
| History of pulmonary hypertension | 2.29 (1.27, 4.12) | 0.0060 | 1.26 (0.66, 2.39) | 0.49 | --- |  |
| Emphysema | 1.38 (0.69, 2.75) | 0.36 | 1.01 (0.48, 2.11) | 0.98 | --- |  |
| Prior hospitalisation | 1.78 (1.17, 2.72) | 0.0072 | 1.19 (0.75, 1.88) | 0.46 | --- |  |
| Distance to enrolling centre, per  10-mile increase | 0.98 (0.95, 1.01) | 0.17 | 0.99 (0.96, 1.03) | 0.63 | --- |  |
| Symptom onset to confirmed diagnosis of IPF at enrolling centre, per 6-month increase | 0.96 (0.89, 1.05) | 0.35 | 0.94 (0.86, 1.02) | 0.15 | --- |  |

Multivariable model included all covariates listed. Adjustment model included patient characteristics selected after performing backwards selection on the multivariable model.

**Table S3.** Associations between patient characteristics and respiratory-related death or lung transplant in univariable models, multivariable model and adjustment model

|  | Univariable models | | Multivariable model | | Adjustment model | |
| --- | --- | --- | --- | --- | --- | --- |
|  | HR (95% CI) | p-value | HR (95% CI) | p-value | HR (95% CI) | p-value |
| Age, years |  | <0.0001 |  | 0.015 |  | 0.019 |
| <62 years, per 5-year increase | 0.52 (0.43, 0.64) |  | 0.53 (0.33, 0.86) |  | 0.51 (0.32, 0.82) |  |
| ≥62 years, per 5-year increase | 1.21 (1.11, 1.31) |  | 1.01 (0.83, 1.24) |  | 1.11 (0.92, 1.34) |  |
| Female sex | 1.07 (0.67, 1.70) | 0.78 | 1.03 (0.61, 1.74) | 0.90 | --- |  |
| Body mass index, per 1-point increase | 0.96 (0.91, 1.00) | 0.063 | 0.95 (0.91, 1.00) | 0.058 | --- |  |
| Private insurance | 0.88 (0.57, 1.36) | 0.56 | 0.95 (0.59, 1.53) | 0.84 | --- |  |
| Current or former smoker | 0.86 (0.56, 1.32) | 0.49 | 0.74 (0.46, 1.20) | 0.23 | --- |  |
| Oxygen use with activity | 4.15 (2.70, 6.38) | <0.0001 | 2.25 (1.28, 3.95) | 0.0049 | 2.13 (1.24, 3.66) | 0.0063 |
| Oxygen use at rest | 5.03 (3.31, 7.64) | <0.0001 | 3.76 (2.06, 6.87) | <0.0001 | 3.33 (1.94, 5.75) | <0.0001 |
| Diagnostic criteria of definite IPF | 1.07 (0.67, 1.69) | 0.79 | 0.94 (0.58, 1.55) | 0.82 | --- |  |
| History of coronary artery disease or congestive heart failure | 1.04 (0.67, 1.60) | 0.87 | 1.35 (0.81, 2.25) | 0.25 | --- |  |
| History of pulmonary hypertension | 2.23 (1.24, 4.02) | 0.0076 | 1.28 (0.68, 2.43) | 0.45 | --- |  |
| Emphysema | 1.22 (0.59, 2.54) | 0.59 | 0.75 (0.34, 1.63) | 0.46 | --- |  |
| Prior hospitalisation | 1.46 (0.95, 2.23) | 0.085 | 0.90 (0.56, 1.45) | 0.66 | --- |  |
| Distance to enrolling centre, per  10-mile increase | 0.99 (0.96, 1.02) | 0.67 | 1.00 (0.97, 1.04) | 0.83 | --- |  |
| Symptom onset to confirmed diagnosis of IPF at enrolling centre, per 6-month increase | 0.96 (0.89, 1.03) | 0.23 | 0.91 (0.84, 0.99) | 0.038 | --- |  |

Multivariable model included all covariates listed. Adjustment model included patient characteristics selected after performing backwards selection on the multivariable model.

**Table S4.** Associations between patient characteristics and respiratory-related death in univariable models, multivariable model and adjustment model

|  | Univariable models | | Multivariable model | | Adjustment model | |
| --- | --- | --- | --- | --- | --- | --- |
|  | HR (95% CI) | p-value | HR (95% CI) | p-value | HR (95% CI) | p-value |
| Age, years |  | <0.0001 |  | 0.13 |  | 0.024 |
| <62 years, per 5-year increase | 0.45 (0.36, 0.57) |  | 0.57 (0.33, 0.98) |  | 0.53 (0.31, 0.90) |  |
| ≥62 years, per 5-year increase | 1.37 (1.25, 1.49) |  | 1.13 (0.91, 1.39) |  | 1.27 (1.05, 1.54) |  |
| Female sex | 0.99 (0.58, 1.68) | 0.96 | 1.00 (0.56, 1.78) | 0.99 | --- |  |
| Body mass index, per 1-point increase | 0.95 (0.90, 1.00) | 0.053 | 0.96 (0.91, 1.01) | 0.095 | --- |  |
| Private insurance | 0.78 (0.48, 1.26) | 0.31 | 0.81 (0.48, 1.36) | 0.42 | --- |  |
| Current or former smoker | 0.86 (0.53, 1.39) | 0.54 | 0.71 (0.41, 1.21) | 0.21 | --- |  |
| Oxygen use with activity | 4.34 (2.67, 7.05) | <0.0001 | 2.37 (1.23, 4.59) | 0.010 | 2.24 (1.19, 4.22) | 0.013 |
| Oxygen use at rest | 5.20 (3.24, 8.34) | <0.0001 | 3.19 (1.64, 6.23) | 0.0007 | 2.90 (1.57, 5.36) | 0.0007 |
| Diagnostic criteria of definite IPF | 1.02 (0.61, 1.72) | 0.93 | 0.94 (0.54, 1.64) | 0.82 | --- |  |
| History of coronary artery disease or congestive heart failure | 1.21 (0.75, 1.95) | 0.44 | 1.42 (0.82, 2.47) | 0.21 | --- |  |
| History of pulmonary hypertension | 2.19 (1.12, 4.27) | 0.022 | 1.12 (0.54, 2.31) | 0.77 | --- |  |
| Emphysema | 1.08 (0.47, 2.51) | 0.86 | 0.72 (0.30, 1.76) | 0.47 | --- |  |
| Prior hospitalisation | 1.80 (1.12, 2.88) | 0.015 | 1.11 (0.66, 1.87) | 0.69 | --- |  |
| Distance to enrolling centre, per  10-mile increase | 0.98 (0.94, 1.01) | 0.21 | 0.99 (0.95, 1.03) | 0.63 | --- |  |
| Symptom onset to confirmed diagnosis of IPF at enrolling centre, per 6-month increase | 0.95 (0.87, 1.03) | 0.20 | 0.91 (0.83, 0.99) | 0.035 | --- |  |

Multivariable model included all covariates listed. Adjustment model included patient characteristics selected after performing backwards selection on the multivariable model.

**Table S5.** Associations between markers of disease severity and death in univariable models and a model adjusted for patient characteristics

|  | Univariable models | | Model adjusted for patient characteristics | |
| --- | --- | --- | --- | --- |
|  | HR (95% CI) | p-value | HR (95% CI) | p-value |
| FVC % predicted, per 10% decrease | 1.38 (1.20, 1.59) | <0.0001 | 1.23 (1.06, 1.43) | 0.0070 |
| DLco % predicted, per 10% decrease | 1.72 (1.46, 2.04) | <0.0001 | 1.34 (1.11, 1.63) | 0.0023 |
| Prior respiratory hospitalisation | 1.83 (1.16, 2.89) | 0.0093 | 1.03 (0.63, 1.67) | 0.91 |
| Number of respiratory-related hospitalisations in past year |  | 0.029 |  | 0.97 |
| 1 vs 0 | 1.71 (1.02, 2.87) |  | 1.06 (0.61, 1.81) |  |
| ≥2 vs 0 | 2.24 (1.02, 4.93) |  | 0.96 (0.42, 2.17) |  |
| CPI, per 5-point increase | 1.45 (1.30, 1.63) | <0.0001 | 1.24 (1.10, 1.41) | 0.0007 |
| GAP stage |  | <0.0001 |  | 0.22 |
| II vs I | 1.49 (0.84, 2.65) |  | 1.27 (0.70, 2.31) |  |
| III vs I | 3.88 (2.08, 7.24) |  | 1.87 (0.90, 3.87) |  |

Adjusted model included the disease severity marker listed plus the following adjustment covariates: age, oxygen use with activity, oxygen use at rest.

**Table S6.** Associations between markers of disease severity and respiratory-related death or lung transplant in univariable models and model adjusted for patient characteristics

|  | Univariable models | | Model adjusted for patient characteristics | |
| --- | --- | --- | --- | --- |
|  | HR (95% CI) | p-value | HR (95% CI) | p-value |
| FVC % predicted, per 10% decrease | 1.67 (1.42, 1.96) | <0.0001 | 1.46 (1.24, 1.72) | <0.0001 |
| DLco % predicted, per 10% decrease | 1.79 (1.50, 2.13) | <0.0001 | 1.38 (1.13, 1.67) | 0.0014 |
| Prior respiratory hospitalisation | 1.91 (1.22, 2.98) | 0.0045 | 1.00 (0.62, 1.61) | 0.99 |
| Number of respiratory-related hospitalisations in past year |  | 0.013 |  | 0.99 |
| 1 vs 0 | 1.76 (1.07, 2.90) |  | 1.02 (0.60, 1.72) |  |
| ≥2 vs 0 | 2.52 (1.14, 5.56) |  | 0.95 (0.42, 2.15) |  |
| CPI, per 5-point increase | 1.58 (1.39, 1.80) | <0.0001 | 1.35 (1.18, 1.56) | <0.0001 |
| GAP stage |  | <0.0001 |  | 0.0055 |
| II vs I | 1.98 (1.05, 3.73) |  | 1.96 (1.01, 3.80) |  |
| III vs I | 5.86 (3.02, 11.4) |  | 3.52 (1.62, 7.65) |  |

Adjusted model included the disease severity marker listed plus the following adjustment covariates: age, oxygen use with activity, oxygen use at rest.

**Table S7.** Associations between markers of disease severity and respiratory-related death in univariable models and model adjusted for patient characteristics

|  | Univariable models | | Model adjusted for patient characteristics | |
| --- | --- | --- | --- | --- |
|  | HR (95% CI) | p-value | HR (95% CI) | p-value |
| FVC % predicted, per 10% decrease | 1.50 (1.28, 1.77) | <0.0001 | 1.32 (1.11, 1.57) | 0.0021 |
| DLco % predicted, per 10% decrease | 1.79 (1.47, 2.18) | <0.0001 | 1.35 (1.08, 1.68) | 0.0078 |
| Prior respiratory hospitalisation | 2.25 (1.38, 3.68) | 0.0012 | 1.21 (0.72, 2.05) | 0.47 |
| Number of respiratory-related hospitalisations in past year |  | 0.0036 |  | 0.78 |
| 1 vs 0 | 2.03 (1.16, 3.56) |  | 1.22 (0.68, 2.19) |  |
| ≥2 vs 0 | 3.00 (1.35, 6.70) |  | 1.20 (0.52, 2.78) |  |
| CPI, per 5-point increase | 1.55 (1.35, 1.77) | <0.0001 | 1.30 (1.12, 1.52) | 0.0009 |
| GAP stage |  | <0.0001 |  | 0.19 |
| II vs I | 1.70 (0.86, 3.34) |  | 1.43 (0.71, 2.89) |  |
| III vs I | 4.72 (2.27, 9.81) |  | 2.15 (0.92, 5.06) |  |

Adjusted model included the disease severity marker listed plus the following adjustment covariates: age, oxygen use with activity, oxygen use at rest.

**Table S8.** Comparison of markers of disease severity between patients with a new diagnosis of IPF and patients referred with a diagnosis of IPF

|  | New diagnosis of IPF  (other ILD or no diagnosis) | Referred with diagnosis of IPF |
| --- | --- | --- |
| Definite IPF by PI | 214 (66.5%) | 210 (71.9%) |
| FVC median (Q1, Q3) | 69.2 (59.7, 80.3) | 69.6 (60.5, 78.9) |
| GAP stage  I  II  III | 81 (27.6%)  164 (55.8%)  49 (16.7%) | 59 (24.3%)  134 (55.1%)  50 (20.6%) |
| CPI median (Q1, Q3) | 53.5 (45.5, 59.6) | 53.0 (46.3, 60.5) |
| Oxygen at rest | 70 (21.5%) | 51 (17.5%) |
| Oxygen with activity | 104 (31.9%) | 104 (35.9%) |

**Table S9.** Joint association between patient characteristics and disease severity markers, and death, respiratory-related death or lung transplant, and respiratory-related death

|  | Death | | Respiratory-related death  or lung transplant | | Respiratory-related death | |
| --- | --- | --- | --- | --- | --- | --- |
|  | HR (95% CI) | p-value | HR (95% CI) | p-value | HR (95% CI) | p-value |
| Age, years |  | 0.018 |  | 0.16 |  | 0.056 |
| <62 years, per 5-year increase | 0.65 (0.40, 1.07) |  | 0.64 (0.40, 1.02) |  | 0.69 (0.40, 1.18) |  |
| ≥62 years, per 5-year increase | 1.29 (1.08, 1.54) |  | 1.13 (0.93, 1.38) |  | 1.28 (1.05, 1.56) |  |
| Oxygen use with activity | 1.43 (0.80, 2.56) | 0.23 | 1.62 (0.93, 2.81) | 0.088 | 1.72 (0.89, 3.29) | 0.10 |
| Oxygen use at rest | 2.15 (1.21, 3.82) | 0.0087 | 2.51 (1.45, 4.36) | 0.0010 | 2.18 (1.17, 4.06) | 0.014 |
| FVC % predicted, per 10% decrease | 1.15 (0.99, 1.35) | 0.075 | 1.38 (1.17, 1.64) | 0.0002 | 1.25 (1.04, 1.49) | 0.018 |
| DLco % predicted, per 10% decrease | 1.27 (1.04, 1.55) | 0.021 | 1.22 (0.99, 1.50) | 0.065 | 1.23 (0.98, 1.55) | 0.079 |

Patient characteristics and disease severity markers were entered into a multivariable model simultaneously.

**Table S10.** Composite physiologic index and GAP stage based on all patients, patients on oxygen at rest and patients not on oxygen at rest

|  | Entire cohort  (n=638) | Oxygen at rest (n=125) | No oxygen at rest (n=513) |
| --- | --- | --- | --- |
| Composite physiologic index | 53.1 (45.6, 59.8) | 62.6 (54.7, 67.0) | 51.7 (44.9, 57.9) |
| GAP stage |  | | |
| I | 143 (26.3%) | 11 (10.5%) | 132 (30.1%) |
| II | 304 (55.9%) | 49 (46.7%) | 255 (58.1%) |
| III | 97 (17.8%) | 45 (42.9%) | 52 (11.8%) |
