# Supplementary material for: Predictors of death or lung transplant after a diagnosis of idiopathic pulmonary fibrosis: insights from the IPF-PRO Registry
Source: Respir Res. 2019 May 30;20:105. doi: 10.1186/s12931-019-1043-9 (PMC6542049; doi:10.1186/s12931-019-1043-9)

**Figure S1.** Kaplan-Meier estimate of time from enrolment in the IPF-PRO Registry to death


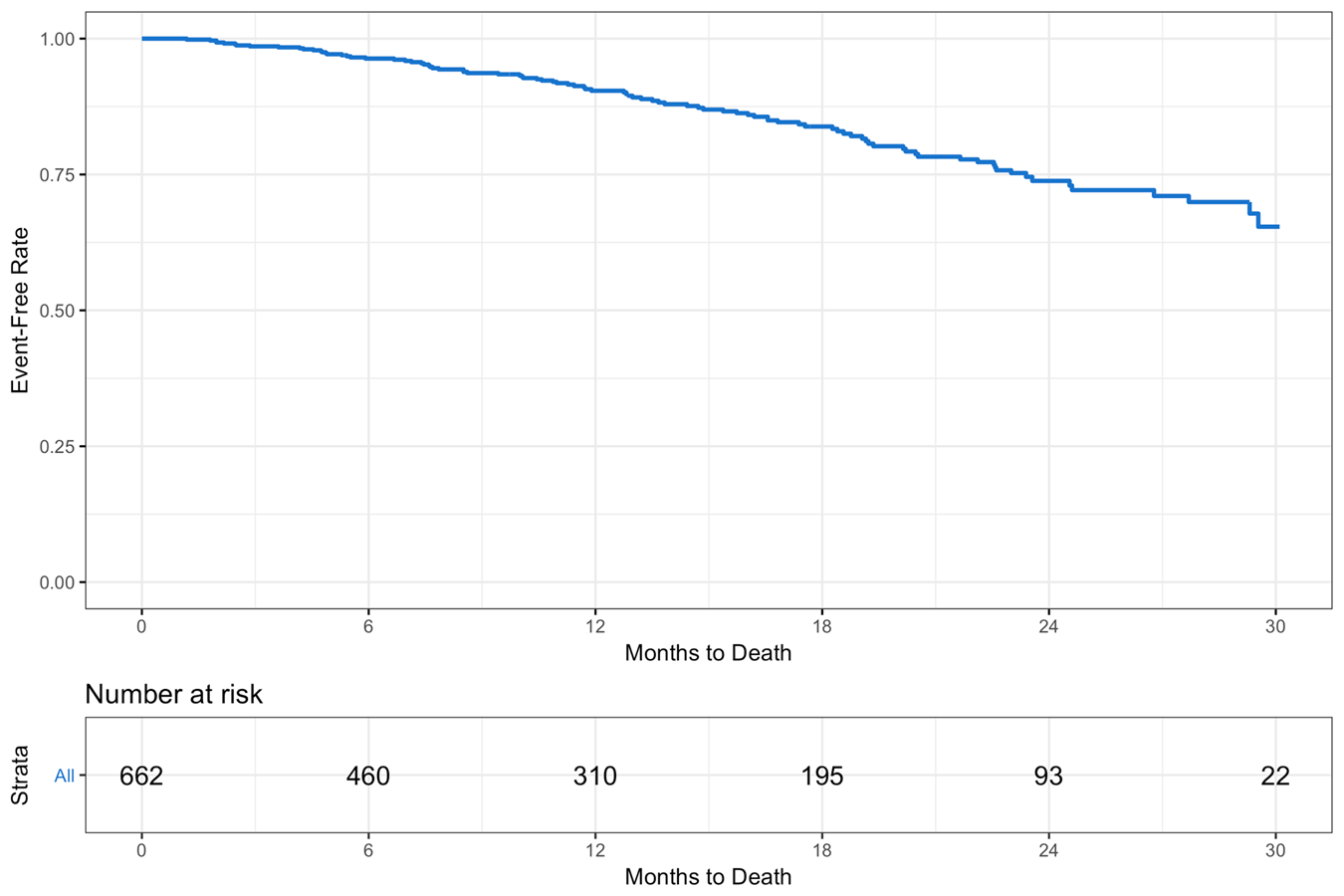


**Figure S2.** Kaplan-Meier estimate of time from enrolment in the IPF-PRO Registry to respiratory-related death or lung transplant


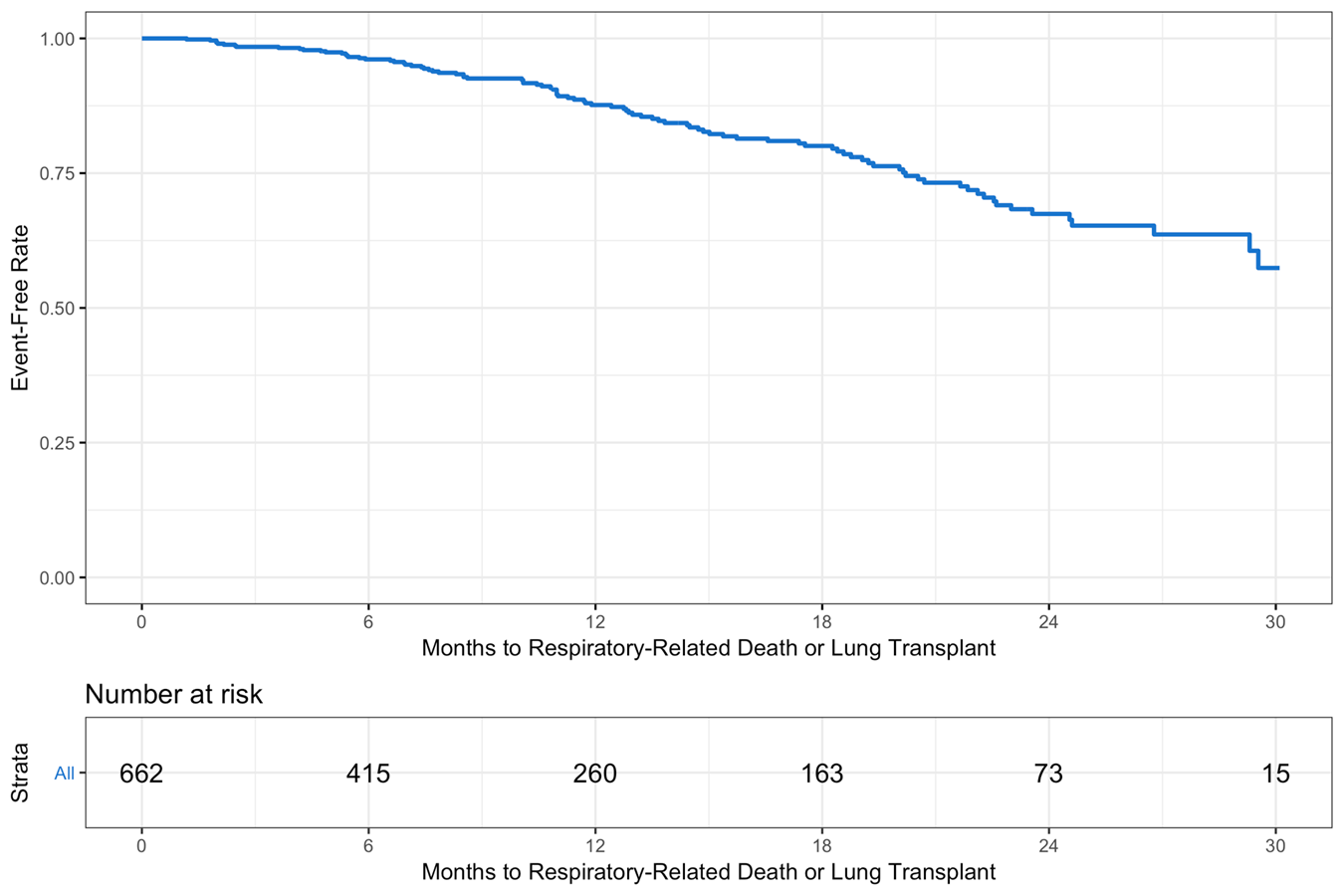


**Figure S3.** Kaplan-Meier estimate of time from enrolment in the IPF-PRO Registry to respiratory-related death


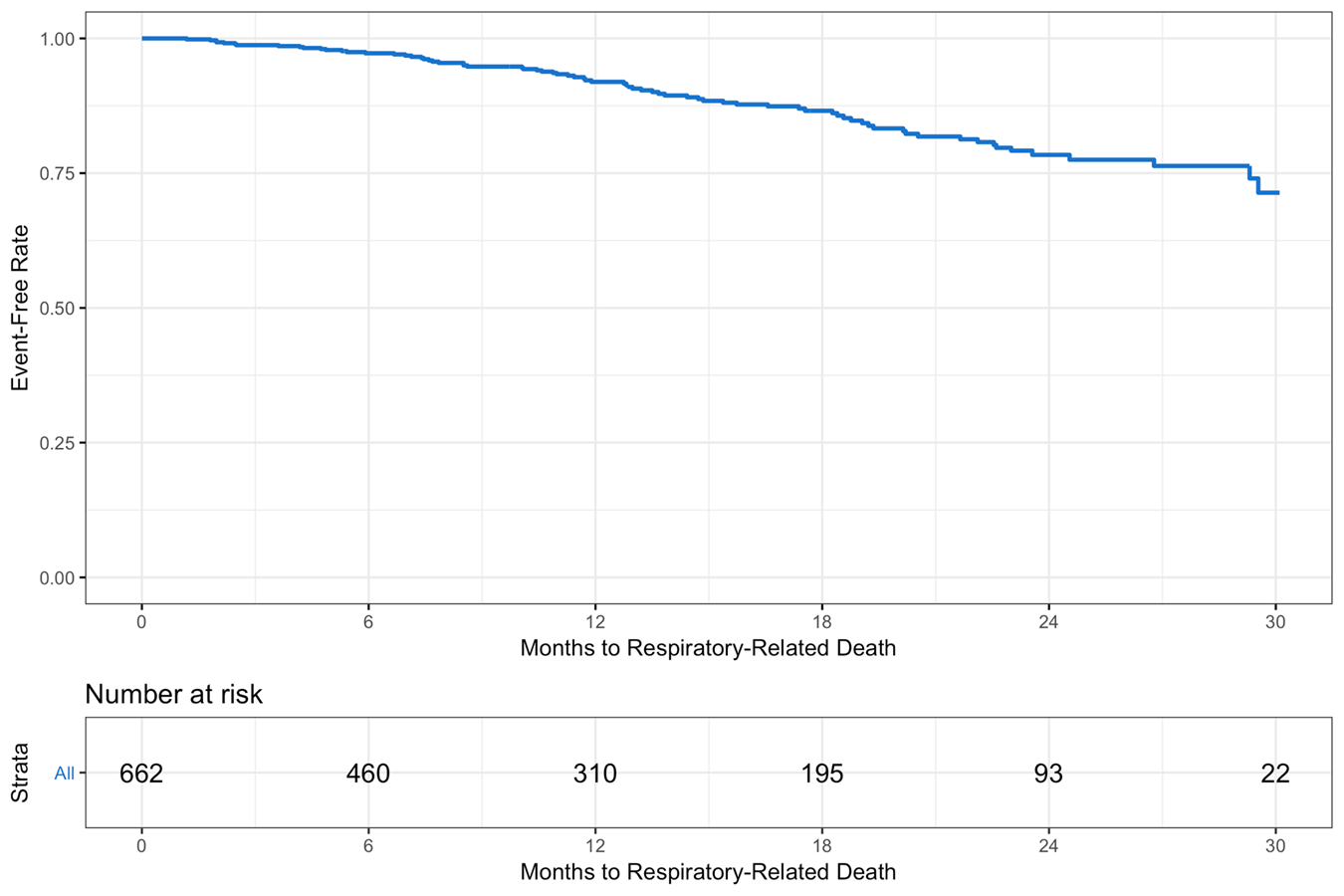

Supplement: Supplementary file 3 — Supplemental figures. (DOCX 299 kb) [file 12931_2019_1043_MOESM3_ESM.docx]
